# Body self-evaluation and physical scars in patients with borderline personality disorder: an observational study

**DOI:** 10.1186/2051-6673-1-2

**Published:** 2014-04-10

**Authors:** Nikolaus Kleindienst, Kathlen Priebe, Elisabeth Borgmann, Sven Cornelisse, Antje Krüger, Ulrich Ebner-Priemer, Anne Dyer

**Affiliations:** Department of Psychosomatic Medicine and Psychotherapy, Central Institute of Mental Health, Medical Faculty Mannheim/Heidelberg University, Germany, J5 Mannheim, D-68159 Germany; Department of Psychology, School of Social Sciences, University of Mannheim, Mannheim, 68131 Germany; Department of Clinical Psychology and Psychotherapy, University of Muenster, Muenster, 48149 Germany; Department of Sport and Sport Sciences, Karlsruhe Institute of Technology, Karlsruhe, 76131 Germany

**Keywords:** Body self-evaluation, Scars, Borderline personality disorder, Childhood sexual abuse, Post-traumatic stress disorder

## Abstract

**Background:**

Data from general psychology suggest that body self-evaluation is linked to self-esteem and social emotions. Although these emotions are fragile in individuals with borderline personality disorder (BPD), body self-evaluation is clearly understudied in BPD research.

**Methods:**

A total of 200 women took part in the study: 80 female BPD patients, and 47 healthy and 73 clinical controls including post-traumatic stress disorder (PTSD) after childhood sexual abuse (CSA). Diagnoses were established through standardised interviews conducted by experienced psychologists. The participants used the Survey of Body Areas to indicate which areas of their own bodies they liked or disliked and to mark the locations of physical scars.

**Results:**

Compared to healthy controls, both BPD patients and patients with PTSD after CSA had a predominantly negative body self-evaluation (Cohen’s d = 1.42 and 1.38, respectively). As indicated by multilevel analyses, scars were related to a negative evaluation of the affected areas in BPD patients, but not in the control groups. Subgroup analyses revealed that the negative body self-evaluation applies to both BPD patients with and without PTSD or reported CSA.

**Conclusions:**

BPD patients show a negative body self-evaluation which is associated with the presence of scars but not with CSA.

## Background

Individuals with borderline personality disorder (BPD) typically suffer from a complex of low self-esteem, negative self-concept and from negative social emotions such as guilt or shame [1–3]. Previous research from general psychology has linked these aspects to body self-evaluation [4–6], but this field is clearly understudied in BPD research. Pioneering data on the body self-evaluation in patients with an established diagnosis of BPD indicate that this patient group reports a more negative body evaluation than healthy controls or psychiatric controls with conditions such as bulimia nervosa [7, 8]. As these studies are published in German and Japanese, respectively, many readers have to rely on studies, which have either been carried out in samples with BPD features [9, 10] or in samples diagnosed with self-rating questionnaires [11, 12]. The data by Muehlenkamp et al. [9, 10] indicate that a negative evaluation of one’s own appearance might aggravate non-suicidal self-injury (NSSI) in adolescents and in patients with eating disorders. The data by Sansone et al. [11, 12] confirm that BPD features are related to less comfort with the own body and suggest a link between a negative body image and social avoidance.

Some of the negative body evaluation associated with BPD may possibly be explained by the presence of visible scars. Up to 90% of BPD patients engage in repetitive NSSI behaviours. The most common forms of NSSI are superficial cutting, scratching, or burning the skin with a cigarette [13, 14], and such injuries often result in numerous scars [15]. Several authors have reported that BPD patients hide these characteristic scars out of anticipation of stigmatisation related to the diagnosis of BPD and self-harm [2, 16]. BPD patients might be especially sensitive to stigmatising labelling processes as (i) they are highly sensitive to social exclusion [17], and (ii) self-stigma has been shown to play a greater role in BPD than in some other psychiatric disorders such as social phobia [18]. On the other hand, social evaluation of NSSI was found to be ambiguous in adolescents [19]; and a self-harm behaviour might foster social affiliation and group identity [20]. This is in line with reports of social contagion with respect to NSSI in BPD groups [21].

The ambiguity in the evaluation of scars might also result from the diversity of intentions related to NSSI. Functional analyses have revealed a wide spectrum of concurrent and alternative motives involved in acts of NSSI such as “reduction of negative feelings” and “self-punishment” [14]. Analysis of the prominence of specific motives indicates that most scars bring up reminders of highly aversive emotions [14, 22]. Consistent with this, BPD patients frequently admit that they sometimes succumb to the urge of cutting against their own will. These might be some of the reasons why many self-injurers have negative emotions towards their scars and often state that they harm the arms or legs “because the wounds can be hidden under clothing” [23]. However, it has also been speculated that some patients “feel a smug pride in [their own] scars” [24] and show them off “like soldiers who wear medals and need to be admired for their bravery in battle” [25]. Trauma research further fosters the concept that the memories related to a specific scar can impact the evaluation of that scar. In a study on victims of interpersonal violence, Weaver et al. [26] found evidence that scars or marks that are reminiscent of unpleasant memories are evaluated more negatively than scars that do not bear any special emotional meaning. In sum, there is some preliminary evidence linking scars in BPD patients to both a negative body self-evaluation and to further issues such as social withdrawal. However, the paucity of specific data suggests a systematic investigation including BPD patients, healthy controls (to assess the magnitude of the problem) and clinical controls (to test for the specificity of the findings).

The first aim of the present study was to quantify differences in body self-evaluation between BPD patients and both healthy and clinical controls. Second, we examined the relationship between body self-evaluation and scars in BPD patients. Finally, we investigated the clinical specificity of this supposed relationship. The clinical controls in this study were patients with post-traumatic stress disorder (PTSD) after childhood sexual abuse (CSA), and with anxiety disorders other than PTSD.

## Methods

### Participants

Study participants were recruited between August 2008 and July 2011. BPD and clinical control patients were recruited from wait-lists at the Department of Psychosomatic Medicine and Psychotherapy at the Central Institute of Mental Health in Mannheim, Germany and through referrals from therapists. In addition, clinical controls and healthy controls were recruited through local newspaper advertisments, and the study was publicised through newspaper articles.

Individuals meeting the following criteria were eligible for the study:

- Female
- Aged between 18 and 65 years
- Fluency in German language
- Either a diagnosis of BPD, PTSD after CSA, or other anxiety disorder (clinical controls), or absence of both BPD and of Axis I disorders (healthy controls)
- No diagnosis of schizophrenia (lifetime) or intellectual disability.

Of 323 prospective subjects assessed for eligibility, 29 chose not to participate, and 47 did not meet the inclusion criteria. Of the 247 who provided written informed consent, 39 did not return the screening questionnaires, and 8 had to be excluded from the analyses as essential data were missing. This resulted in a final sample of 200 evaluable subjects. Particpants were classified as being in one of four groups: (i) BPD according to DSM-IV, (ii) PTSD according to DSM-IV, but no BPD, (iii) anxiety disorders according to DSM-IV, but no PTSD and no BPD, and (iv) healthy controls with neither an Axis I disorder nor BPD. The study was approved by the responsible ethics board of the Medical Faculty Mannheim at Heidelberg University.

### Assessments

#### Diagnostic procedure and questionnaires

Diagnostic criteria for BPD were assessed using the International Personality Disorder Examination IPDE; [27]. Anxiety disorders including PTSD were diagnosed according to the Structured Clinical Interview for DSM-IV Axis I (SCID I); [28]. Healthy controls were screened for psychiatric disorders using the SCID I and the BPD section of the IPDE. All interviews were assessed by experienced clinical psychologists who were trained to administer these instruments.

The clinical assessment included self-rating instruments for severity of borderline symptomatology Borderline Symptom List [BSL-23]; [29] and PTSD symptoms Posttraumatic Stress Diagnostic Scale [PDS]; [30] as well as for childhood trauma including sexual abuse Childhood Trauma Questionnaire [CTQ]; [31]. Following the cutoff-scores recommended by Bernstein et al. [31], patients with a score ≥ 13 on the subscale Sexual Abuse of the CTQ were considered to be victims of severe CSA, while patients with a score < 6 on this subscales were considered to have no history of CSA.

#### Assessment of body evaluation and of scars

Body self-evaluation was based on the Survey of Body Areas (SBA), which is similar to the widely used German Pain Questionnaire developed by the German section of the International Association for the Study of Pain [32]. The English version of this newly developed open source assessment instrument is described in detail at http://www.sci-mate.org/item.php?id=19606. Briefly, respondents are asked to colour in a pair of female standardised drawings (i.e., front and back), using different coloured crayons to indicate which of their own body areas they either like or dislike. Green is used to code for positively rated areas and red for negatively rated areas; neutral areas are left unmarked. For a numerical evaluation, the standardised drawing is divided into 43 areas. Positively rated areas are coded with +1; negatively rated are coded with −1; and neutral and ambiguous areas (rated both positively and negatively) are coded with 0. This allows the calculation of several indices, including the number of positively and negatively rated areas (both ranging from 0 to 43) and mean values (ranging from −1 to +1), which are calculated as unweighted arithmetic means of the 43 body areas.

To assess the reliability of the SBA, several key indices were tested. To assess retest reliability, 20 patients with PTSD after CSA were asked to fill in the SBA twice, with a minimum of 7 days between the two assessments. Reliability was high for all of the key indices, including the positively rated areas (Pearson’s r = .884), the negatively rated areas (r = .960), and the total numbers of areas with scars (r = .870). Furthermore, convergent validity was established from a sub-sample including 78 patients with BPD, 36 patients with PTSD after CSA but without BPD, and 41 healthy controls. As expected, key indices of the SBA were highly correlated with body image as assessed by the Appearance Evaluation and the Body Areas Satisfaction Scales of the Multidimensional Body-Self Relations Questionnaire (MBSRQ); [33]. The correlations between the number of positively evaluated areas and the Appearance Evaluation and the Body Areas Satisfaction Scales were .623 and .572, respectively. Conversely, the correlations between the number of negatively evaluated areas and the Appearance Evaluation and the Body Areas Satisfaction Scales were -.516 and -.588 (all p-values < .001).

In this study, we provided a second identical pair of standardised drawings (i.e., front and back) in which participants were asked to sketch the location of any scars on their own bodies. Besides establishing the number and distribution of scars, the information from these second standardised drawings was matched to the information on evaluation of the respective body areas from the first standardised drawings. As regions that are typically affected by scars (e.g., forearms) were expected to be rated differently from regions that are rarely affected by scars (e.g., eyes), adjusted scores were used for analyses addressing the potential relation of scars and the evaluation of the respective body areas. To this end, the actual rating of the body area *j* of participant *i* (*area*_*ij*_) was centred at the average evaluation of the respective body area in the group of healthy controls .

#### Statistical evaluation

Mean body ratings across the four groups were compared by both a one-way analysis of variance (ANOVA) and an analysis of covariance (ANCOVA) that adjusted for age and for the total number of scars. Pair-wise post hoc comparisons were Bonferroni-adjusted. For each group, Bonferroni-adjusted one-sample t-tests were used to test whether the average self-evaluation would differ significantly from a neutral score of 0. This procedure allowed us to test whether the BPD patients in our sample typically had predominantly positive or negative evaluations of their own bodies. However, as the majority of BPD patients have co-occurring anxiety disorders including PTSD, a more sophisticated approach was necessary to disentangle the relations between the diagnoses under investigation and the body evaluation. Accordingly, we used multilevel analyses to simultaneously investigate the relation between body evaluation and several psychiatric diagnoses within one individual, and to analyse the relation between scars and body evaluation. Within these hierarchical linear models, the evaluation of a specific body area by a specific person (*y*_*ij*_) was modelled by both variables on the level of persons (Level 2 predictors) and variables on the level of body areas (Level 1 predictors). Level 2 included the main effects of the three psychiatric diagnoses under investigation (BPD, PTSD, and other anxiety disorders) and the two covariates to be controlled for (age and the total number of scars). Level 1 included both the main effect of scars (yes/no) and the interactions between diagnoses and scars. Parameters were estimated using restricted maximum likelihood estimates. The method by Kenward-Roger was used to estimate the standard errors of fixed effects. Marginal residuals were plotted against the predicted means to check for a potential misspecification, and Q-Q plots were used to check for normality of the marginal residuals.

Statistical evaluation further included the extension of Fisher’s exact test to polytomous contingency tables [34] and Pearson’s correlation. Post-hoc comparisons and subgroup analyses were carried out to narrow down the findings of the primary analyses.

Cohen’s d with pooled standard deviation was used as an estimate of between-group effect-sizes for continuous data. Two-tailed p-values ≤ 0.05 were considered statistically significant. All statistical analyses were carried out using SAS version 9.2.

## Results

### Participants

The total number of participants (*n* = 200) comprised 80 patients with BPD; 36 patients with PTSD after CSA but without BPD; 37 patients with anxiety disorders other than PTSD (mostly social phobia: 55.3%, panic disorder with agoraphobia: 21.1%, panic disorder without agoraphobia: 15.8%) and no BPD; and 47 healthy controls without any psychiatric disorder. All participants were adult females (range: 18 to 59 years; mean: 33.50 ± 10.57). As shown in Table 1, the average age was somewhat higher in the clinical control groups compared to the BPD group and the healthy control group, resulting in a significant difference (*F* = 2.73, *df* = 3,189, *p* = 0.045) across the four groups. For further patient characteristics at study entry see Table 1.

**Table 1:** **Patient characteristics (mean ± standard deviation)**

	HC	BPD	PTSD	Other AD	p
Age	31.23 ± 11.52	32.40 ± 9.68	36.75 ± 9.24	35.84 ± 11.72	0.045
Body Mass Index	24.74 ± 5.65	28.74 ± 8.62	26.33 ± 8.70	25.63 ± 10.23	0.154
Borderline Symptom List (BSL-23)	0.25 ± 0.34	2.37 ± 0.94	1.61 ± 0.87	0.93 ± 0.74	< 0.001
Sexual Abuse*	5.49 ± 1.97	13.46 ± 7.94	19.11 ± 5.89	6.56 ± 4.89	< 0.001
Physical Abuse*	5.53 ± 2.06	12.09 ± 6.77	11.69 ± 4.99	7.33 ± 4.16	< 0.001
Emotional Abuse*	7.27 ± 2.96	18.72 ± 5.87	18.97 ± 4.12	10.44 ± 5.75	< 0.001
Emotional Neglect*	8.23 ± 4.26	19.05 ± 5.34	20.27 ± 3.79	12.89 ± 6.61	< 0.001
Physical Neglect*	6.39 ± 2.66	12.26 ± 5.75	12.17 ± 3.23	7.33 ± 3.76	< 0.001
Experience of Inconsistence*	4.35 ± 2.19	11.28 ± 3.99	11.57 ± 3.22	7.39 ± 3.78	< 0.001
Number of Scars**	2.45 ± 2.66	34.22 ± 52.42	18.50 ± 44.24	8.00 ± 18.85	< 0.001

*Subscales from the CTQ.

**The number of scars only refers to patients who reported a numerical number. As for the skewed distribution a non-parametric test has been used to compare the numbers across the four groups.

As the focus of this article is the description of BPD patients, BPD patients with anxiety disorders were included in the BPD group. On average, 3.5 co-occurring current Axis I disorders, mostly PTSD (47.5%) and major depressive disorder (38.8%) were observed in this broad group of BPD patients. The average number of co-occurring Axis I disorders in both clinical control groups was 2.2: for PTSD patients, these were mostly major depressive disorder (72.2%) and anxiety disorders (38.9%); and for patients with other anxiety disorders, they were mostly major depressive disorder (39.5%) and other anxiety disorders (39.5%). There were more victims of severe CSA in the BPD group (58.1%) and the PTSD group (88.9%) than in the group with other anxiety disorders (8.1%) and the healthy controls (6.4%).

### Evaluation of body areas

**Figure 1:**
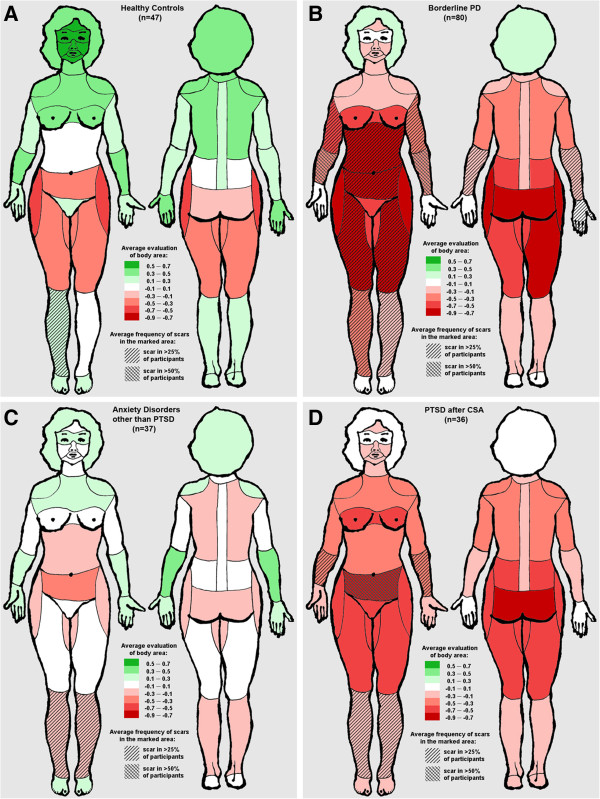
**Average evaluation of body areas and frequency of scars in A: healthy participants, B: Borderline Personality Disorder, C: Anxiety Disorders other than PTSD, D: PTSD after CSA.**

Figure 1 indicates that BPD patients show different patterns and overall evaluation of the own body than the other groups. While green colours (denoting a positive mean evaluation) are seen in healthy participants and in patients with anxiety disorders other than PTSD, red (denoting a negative mean evaluation) prevailed in patients with BPD. However, this finding does not seem to be specific to BPD as much of the negative evaluation of the own body is similar to the group of patients with PTSD after CSA. Statistical testing supported this interpretation of Figure 1. The mean evaluation of body areas was significantly different across the four diagnostic groups (*F* = 24.06, *df* = 3,191, *p* < 0.0001). This result was fully confirmed when including age and the total number of scars as covariates (*F* = 12.70, *df* = 5,167, *p* < 0.0001 for the overall model and *F* = 20.69, *df* = 3,167, *p* < 0.0001 for the effect of group). For both the analysis of variance and the analysis of covariance, Bonferroni-corrected post hoc analyses indicated four significant differences: (i) between BPD patients and healthy controls (Cohen’s d = 1.42), (ii) between BPD patients and patients with anxiety disorders other than PTSD (d = 1.00), (iii) between PTSD patients and healthy controls (d = 1.38), and (iv) between PTSD patients and patients with anxiety disorders other than PTSD (d = 0.95).

**Figure 2:**
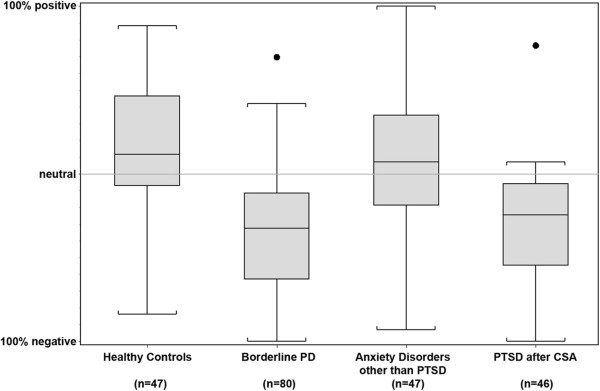
**Body self-evaluation across the diagnostic groups.** Values range from −1 (= 100% negative) to +1 (= 100% positive). Medians and quartiles are marked by the lines of the boxes. Outliers are marked by a dot.

As indicated in Figure 2, the average body rating among healthy controls was significantly more positive than the point of neutral evaluation (0.17 ± 0.35, *p* = 0.002). In contrast, the average rating in the group of BPD patients was clearly negative (−0.34 ± 0.37, *p* < 0.001). The ratings in the clinical control groups were in between: Those of PTSD patients were generally negative (−0.24 ± 0.40, *p* < 0.001), while no trend was seen for the patients with other anxiety disorders (0.06 ± 0.43, *p* = 0.42).

To further illustrate these findings, we calculated percentages of participants in whom the positively rated body areas outnumbered the negatively rated. In 61.7% of healthy controls, most body areas were rated as positive, compared to 11.3% of patients with BPD, 48.7% of the clinical control patients with other anxiety disorders, and 11.1% of patients with PTSD after CSA.

### Presence of scars

The average percentage of body areas containing at least one scar was significantly different between the four diagnostic groups (*p* < 0.001). Of the 43 areas defined by the SBA, BPD patients reported an average of 20.4% as having a scar. The percentages for the other three groups were 14.0% (PTSD after CSA), 7.6% (other anxiety disorders), and 6.4% (healthy controls). As illustrated in Figure 1, the most frequently affected areas in the BPD group were the inner sides of the left and right forearms (68.4% and 55.3%, respectively), followed by the inferior region of the belly (47.4%) and the front side of the left (40.8%) and right (36.8%) upper arms. Among PTSD patients, the regions with the highest incidence of scars were the inferior region of the belly (55.9%), the inner sides of the left and right forearms (32.4% for either side), and the right and the left shins (29.4% and 26.5%, respectively). Among patients with other anxiety disorders, the highest incidences were reported for the left and right shins (29.5% and 25.8%, respectively), followed by the frontal sides of the right arm and forearm (20.0% for both regions). Healthy controls mostly reported scars on the right and the left shins (31.0% and 23.8%, respectively), the upper and inferior regions of the belly (19.0% and 14.3%, respectively), and the hips (11.9%).

### Subgroup analyses for BPD patients with and without PTSD or reported CSA

As a high percentage of BPD patients had either PTSD or elevated scores of CSA, we decided to split the BPD group into (a) a group of BPD patients without PTSD and with no documented CSA (i.e. a score of 5 on the subscale Sexual Abuse of the CTQ; n = 17), and (b) a group of BPD patients with PTSD and/or CSA (i.e. a score ≥ 6 on the subscale Sexual Abuse of the CTQ; n = 63). The corresponding subgroup analyses allowed to investigate whether the predominantly negative evaluations in BPD patients and the negative relation between these evaluations and the presence of scars seen in these patients might be related to PTSD or CSA.

These post-hoc analyses revealed a clearly negative body rating in either of the subgroups (−0.30 ± 0.31, p = 0.001 for BPD patients without PTSD and without CSA, and −0.35 ± 0.39, p < 0.001 for BPD patients with PTSD and/or CSA). Accordingly, only a minority of patients from either subgroup rated more body areas positively than negatively (5.9% of the BPD patients without PTSD or CSA, and 12.9% of the BPD patients with PTSD and/or CSA). In either subgroup the predominantly negative ratings were found in body areas with at least one scar as well as in body areas unaffected by a scar, although the adjusted evaluations of body areas were even more negative. The adjusted differences between affected and unaffected areas were similar for BPD without PTSD or CSA (0.19 ± 0.40) and BPD patients with PTSD or CSA (0.21 ± 0.31).

In sum, subgroup analyses yielded quite similar results for BPD patients with and without PTSD or a history of CSA.

### Multilevel analyses

Multilevel analyses confirmed that body self-evaluation was negatively related to the presence of either BPD or PTSD after CSA, but not to other anxiety disorders. The main effects were significant for the diagnoses of BPD (*β* = −0.264 ± 0.064, p < 0.001) and of PTSD (*β* = −0.283 ± 0.066, p < 0.001) but not for other anxiety disorders (*β* = 0.022 ± 0.063, p = 0.72). As shown in Table 2, the covariates (age and total number of scars) were not significantly related to the body evaluations within this model.

**Table 2:** **Parameter estimates from the hierarchical linear model predicting the evaluation of body areas from diagnoses and the presence of scars**

Effect	Parameter estimate ± Standard error	***t***	***p***
Intercept	−0.0248 ± -0.1077	−0.23	0.818
*Covariates*			
Age	−0.0019 ± 0.0030	−0.63	0.529
Total number of scars	−0.0443 ± 0.2054	−0.22	0.829
*Main effects*			
Presence of a scar	−0.1051 ± 0.0569	−1.85	0.067
Diagnosis of BPD	−0.2641 ± 0.0639	−4.13	< 0.001
Diagnosis of PTSD	−0.2831 ± 0.0664	−4.26	< 0.001
Diagnosis of other anxiety disorders (AD)	−0.0221 ± 0.0611	−0.36	0.718
*Interactions*			
(Presence of a scar)*(Diagnosis of BPD)	−0.1387 ± 0.0639	−2.17	0.032
(Presence of a scar)*(Diagnosis of PTSD)	0.0909 ± 0.0640	1.42	0.159
(Presence of a scar)*(Diagnosis of other AD)	0.0108 ± 0.0633	0.17	0.865

No significant overall association between the presence of at least one scar and the evaluation of the respective body area emerged from the multilevel analyses (*β* = −0.105 ± 0.061, p = 0.067; see Table 2). However, the interaction between the presence of scars and the diagnosis of BPD predicted significantly lower ratings of body areas (*β* = −0.139 ± 0.064, p = 0.032). This indicates that scars are related to a negative evaluation of the affected body areas in BPD patients. This finding was specific for the BPD group; the interactions between scars and the other diagnoses were not significant (p-values >0.15).

**Figure 3:**
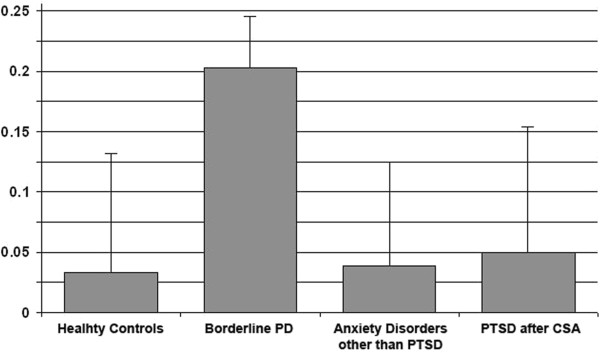
**Differences between adjusted evaluations of body areas affected and not affected by at least one scar (means and standard errors).**

The specificity of the relation between the presence of scars and the evaluation of body areas with or without scars in BPD patients is further illustrated in Figure 3. In the healthy controls and in both clinical control groups, the area-adjusted body evaluations were not significantly related to the presence of scars (all p-values > 0.50). In contrast, BPD patients gave substantially higher evaluations to body areas not affected by a scar than to those that were affected, even when adjusting for the respective areas (0.203 ± 0.328, p < 0.001). The adjustments account for differences in evaluations of areas typically unaffected by scars, such as the backbone, vs. more frequently affected areas, such as arms and legs.

## Discussion

Three major findings emerge from this study. First, in contrast to healthy participants BPD patients had predominantly negative ratings for most of their body areas. This dominance of negative ratings was also seen in patients with PTSD after CSA but not in patients affected by other anxiety disorders. Second, we found that the negative body evaluation in patients with BPD was largely independent of CSA. Third, in BPD patients, the negative evaluation of body areas was significantly related to the presence of scars. This relation between scars and negative evaluation of affected areas was not seen in any of the other groups.

These findings corroborate and extend previously published results. While Sansone et al. [11, 12] and Haaf et al. [7] used standardised body image questionnaires to assess body self-evaluations, we used an approach in which participants were asked to mark all positively or negatively evaluated body areas. Both strategies provide evidence for negative body evaluations in BPD patients. The numbers from our study (notably, the large between-group effect size of d = 1.42 that separated BPD patients from healthy controls, and the low percentage of just 11% in the BPD group that had a generally positive rating of their body) help to better evaluate the magnitude of negative body self-evaluation in BPD patients.

According to our data the negative body self-evaluation and the relation of scars and a negative body evaluation is also seen in BPD patients without PTSD or reported history of CSA. This is in line with the findings from Haaf et al. [7] indicating that childhood trauma did not predict body image in BPD patients. However, our study is the first to establish a link between scars and a negative evaluation of the affected body areas in BPD patients. The specificity of this connection might have different reasons and cannot be explained from our data. Some patients might tend to injure body areas they dislike, while some patients might dislike body areas that have scars resulting from NSSI. Obviously, these two possibilities might also co-occur in one patient. One possibility that should be kept in mind is that characteristic scars resulting from NSSI might contribute to labelling processes as being mentally ill. In a group of patients who is prone to shame [2] and to deficits in close relationships [35] visible scars might be one of the elements in a complex which includes a negative body self-evaluation, perceived social exclusion, dysfunctional coping strategies such as NSSI resulting in scars, which in turn might aggravate a negative body self-evaluation. However, the postulated causal attributions are still to be established. While these possibilities would be in line with our data, our study lends no support to the assumption that these patients feel pride in their scars or want to be admired for them [24, 25].

Several limitations need to be considered when interpreting these results. (i) It would have been desirable to know more about the nature of the scars. It seems plausible that the scars in the BPD patients were generally more visible, and had more often resulted from intentional injuries than those in the control groups. However, as this has not been assessed in our study, all interpretations related to the nature of scars remain speculative. (ii) A related limitation has to do with the choice of clinical controls; patients with PTSD after CSA or with other anxiety disorders. In general, scars of these patients are likely to be less noticeable than say, in individuals whose PTSD was brought on by an accident. To better disentangle different components that might contribute to the evaluation of body areas – both with and without scars – this specific group of clinical controls was not sufficient. While our data give a first hint that different groups of anxiety disorders might differ with respect to body self-evaluation and to a potentially mediating role of scars, a more systematic evaluation of anxiety patients is a matter for future research. (iii) With respect to the external validity of our findings on BPD patients, it should be kept in mind that our sample was confined to females recruited at a specialized university setting. Accordingly, the findings need to be replicated with more diverse samples before they can be generalized to all BPD patients. (iv) A further limitation relates to the sample size, especially in the groups of clinical controls. As indicated by post hoc power analyses, our study was underpowered to detect small between-group differences. (v) As already emphasised, our study was strictly observational; accordingly, it must be acknowledged that all inferences about causal relationships are largely speculative.

Despite these limitations, it may be concluded that at least treatment seeking female BPD patients show a negative evaluation of their body, and this negative evaluation is largely independent of a history of CSA. In addition, this negative evaluation is partially related to the presence of scars.

Future research might elaborate on our finding that the negative body self-evaluation seen in BPD patients is also found in patients without co-occurring PTSD or a history of CSA. At this respect, several types of studies are warranted. Cross-sectional studies could seek to narrow down the link between BPD and a negative body evaluation by excluding confounders such as eating disorders and depression. Longitudinal studies would be needed to establish a link between body-related issues (which are a normal transient phenomenon during adolescence) and the development of BPD. For instance, it would be highly interesting to study whether a persistently negative body self-evaluation is a frequent precursor of BPD. And finally, a development and evaluation of body-oriented treatment modules would be highly desirable.

## Conclusions

Our study indicates that most BPD patients have predominantly negative feelings towards their own body. This negative body self-evaluation was independent from CSA experience, but significantly related to the presence of scars.

As negative self-evaluation of the body is likely to affect well-being, social interaction, and self-esteem, which are already impaired in BPD, our data suggest a systematic evaluation of treatment modules specifically addressing body-related issues in BPD.

From a nosological perspective, we were surprised that experience of CSA and/or PTSD was unrelated to body self-evaluation in BPD. While this finding does not allow for firm conclusions, it invites systematic research addressing the hypotheses that body-related issues are amongst the core symptoms of BPD.

## Abbreviations

BPD: Borderline personality disorder
PTSD: Posttraumatic stress disorder
CSA: Childhood sexual abuse
NSSI: Non-suicidal self-injury
IPDE: International personality disorder examination
SCID I: Structured clinical interview for DSM-IV Axis I
BSL-23: Borderline symptom list
PDS: Posttraumatic stress diagnostic scale
CTQ: Childhood trauma questionnaire
SBA: Survey of body areas
MBSRQ: Multidimensional body-self relations questionnaire
ANOVA: Analysis of variance
ANCOVA: Analysis of covariance
